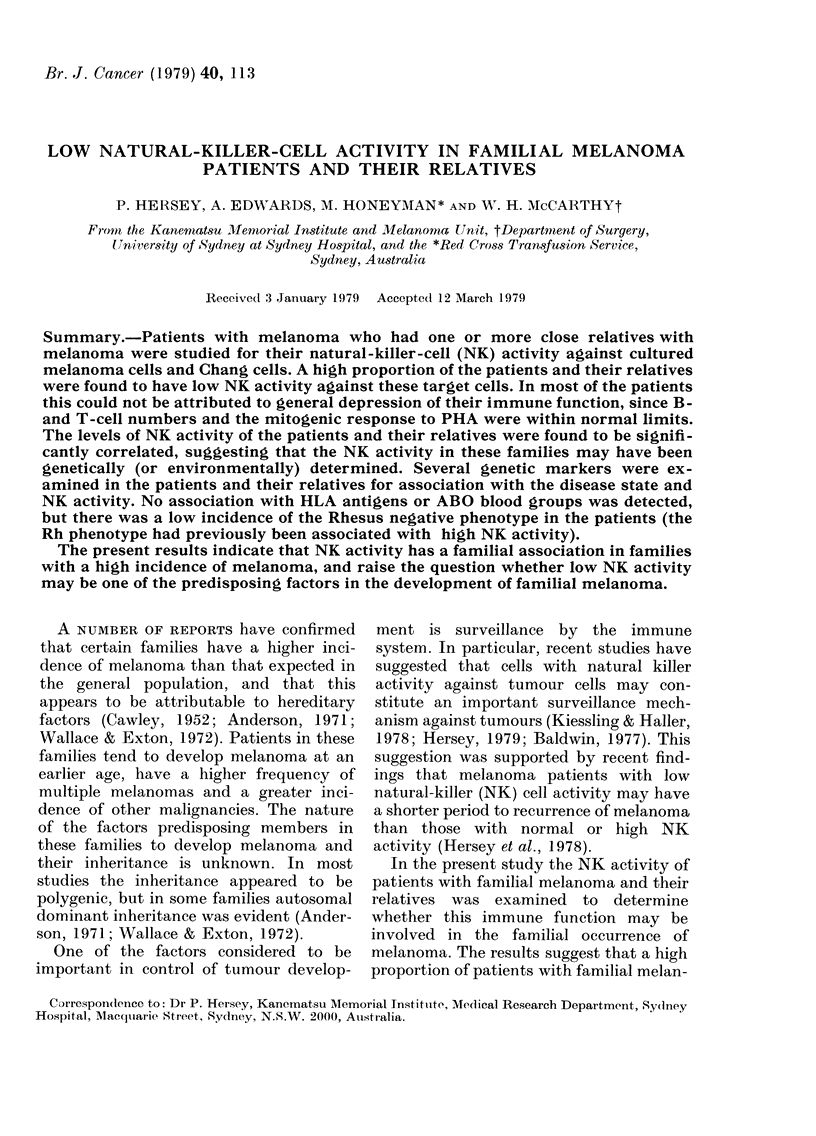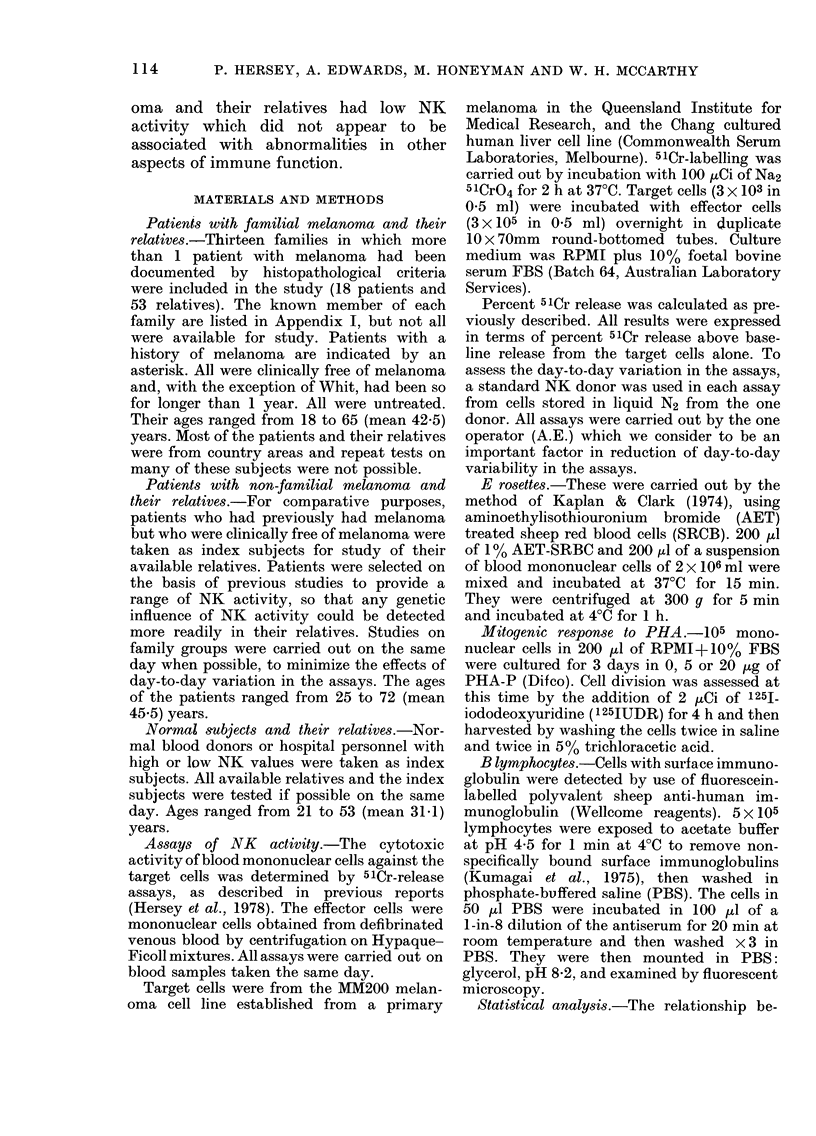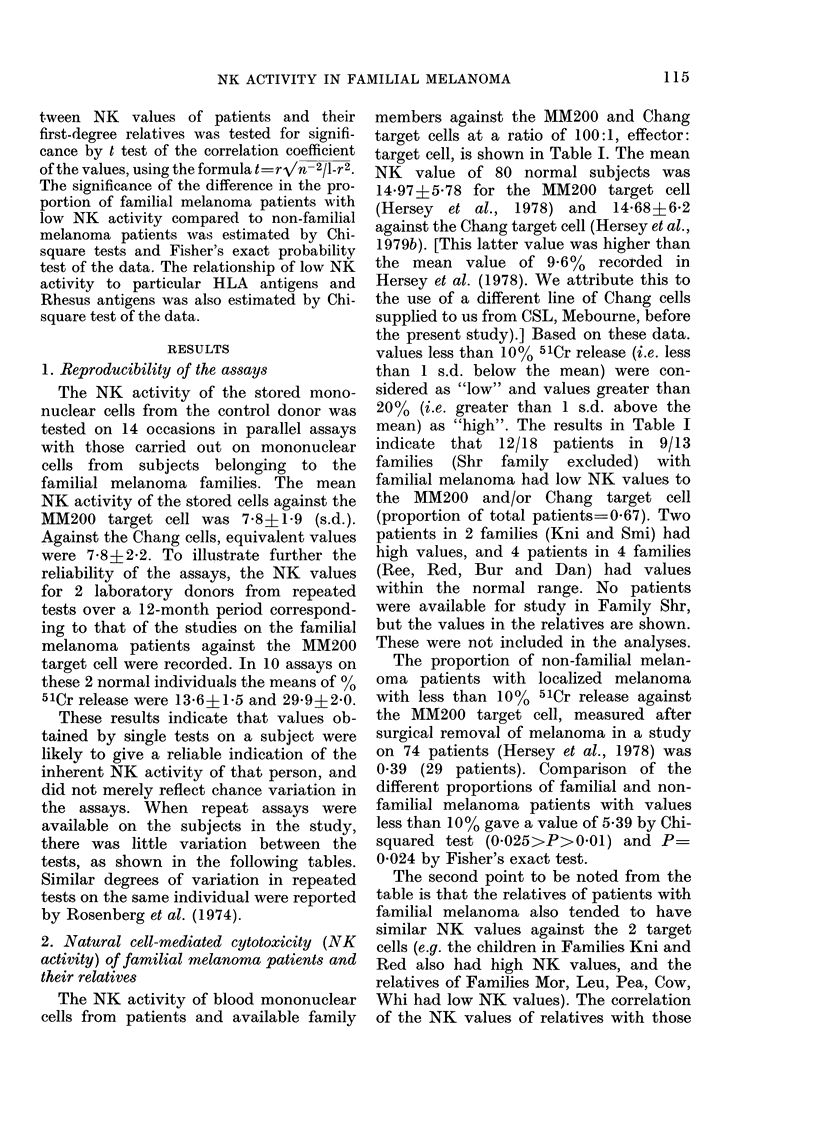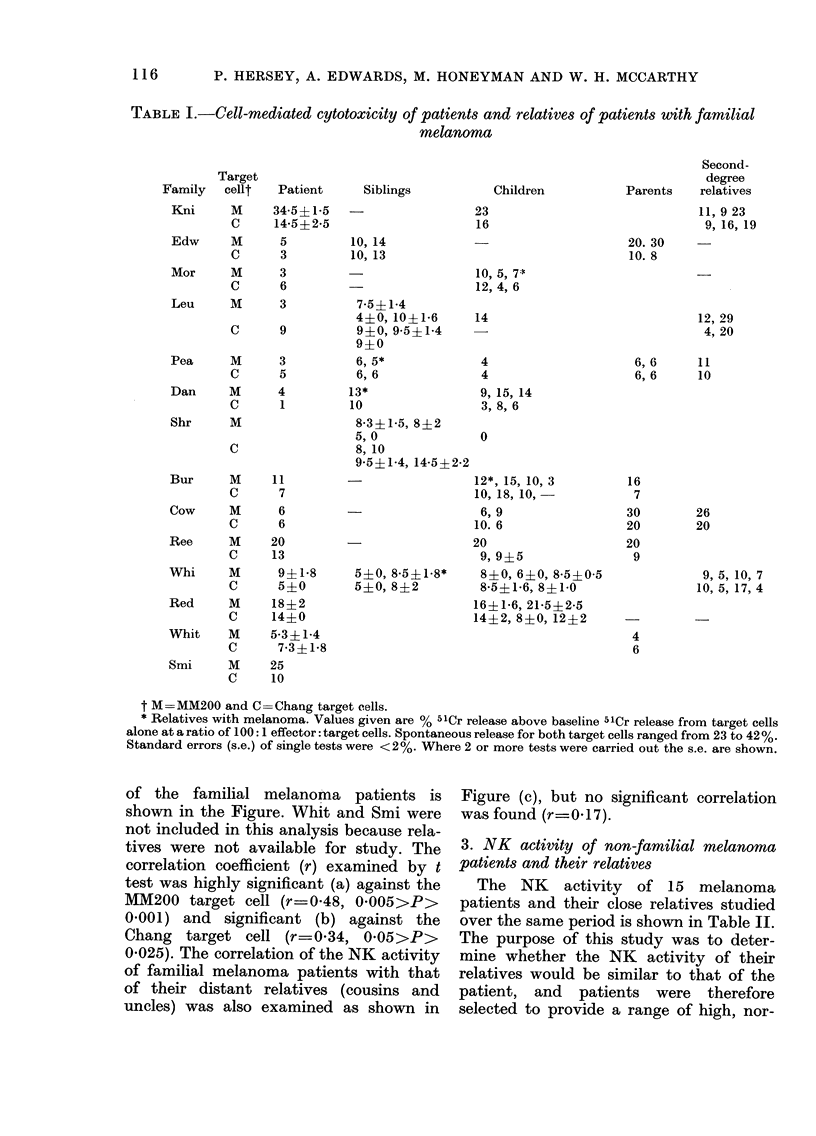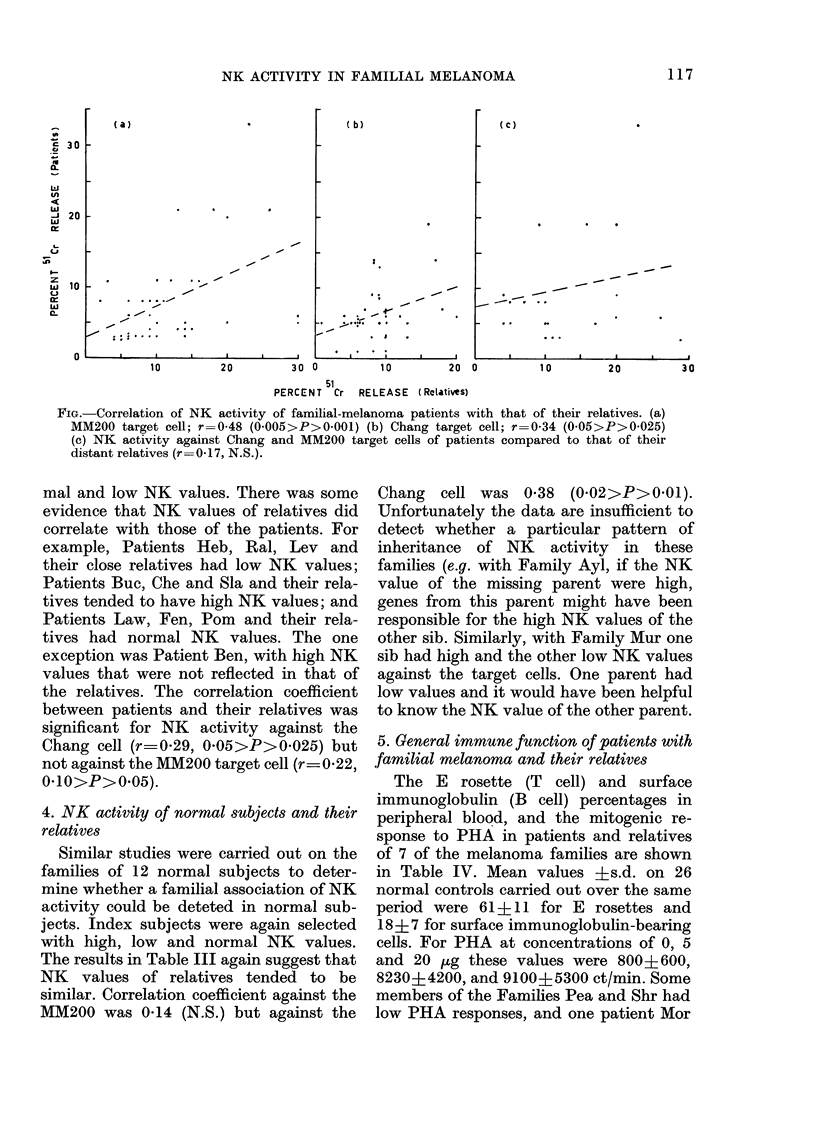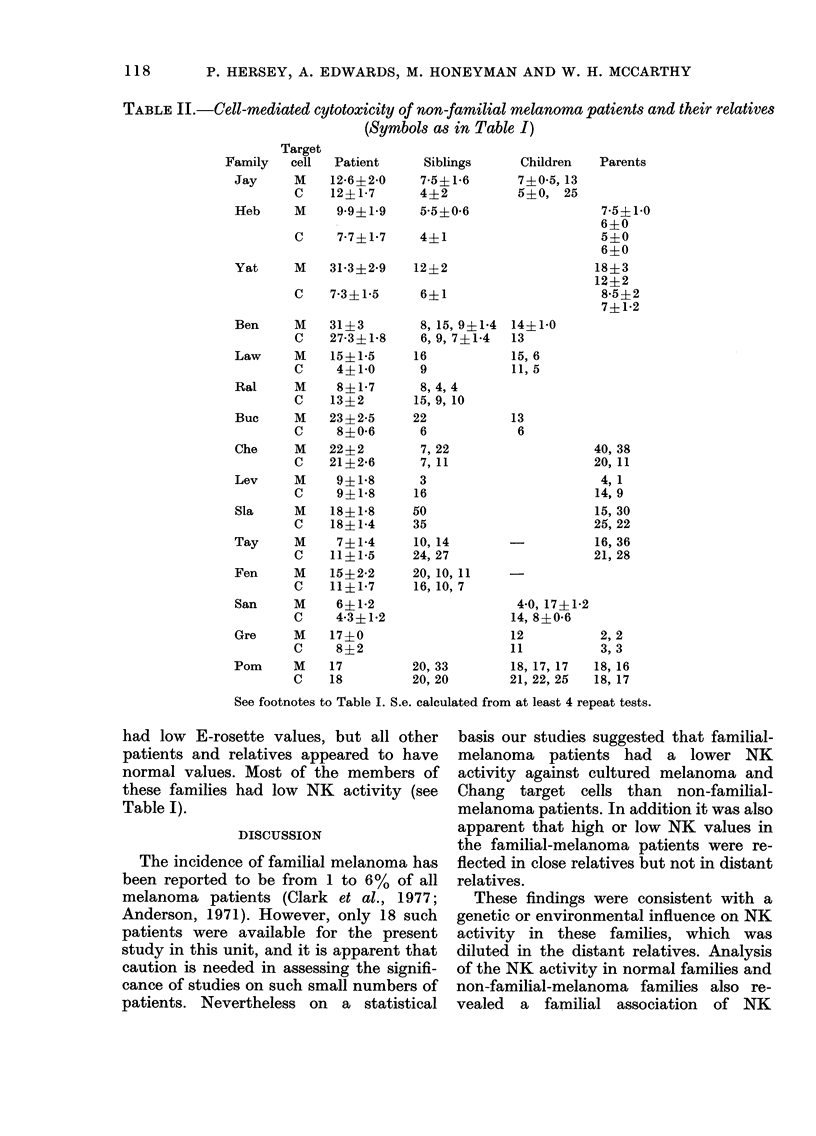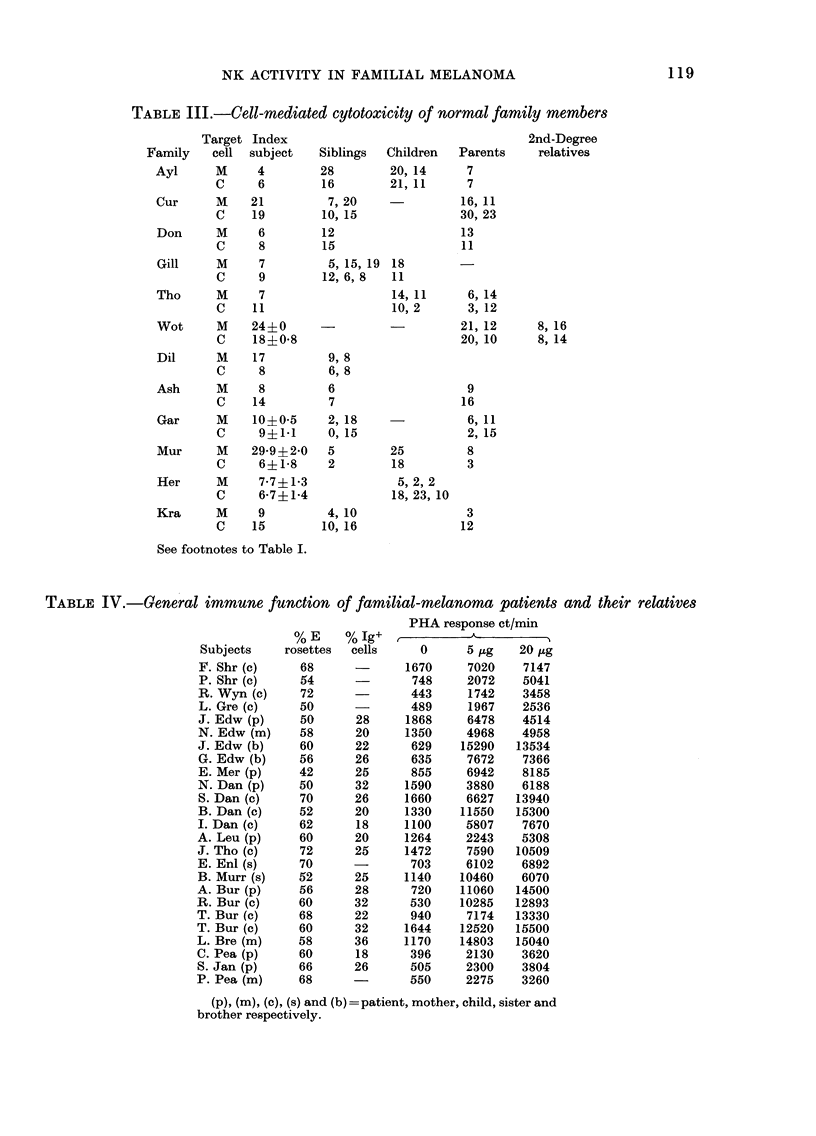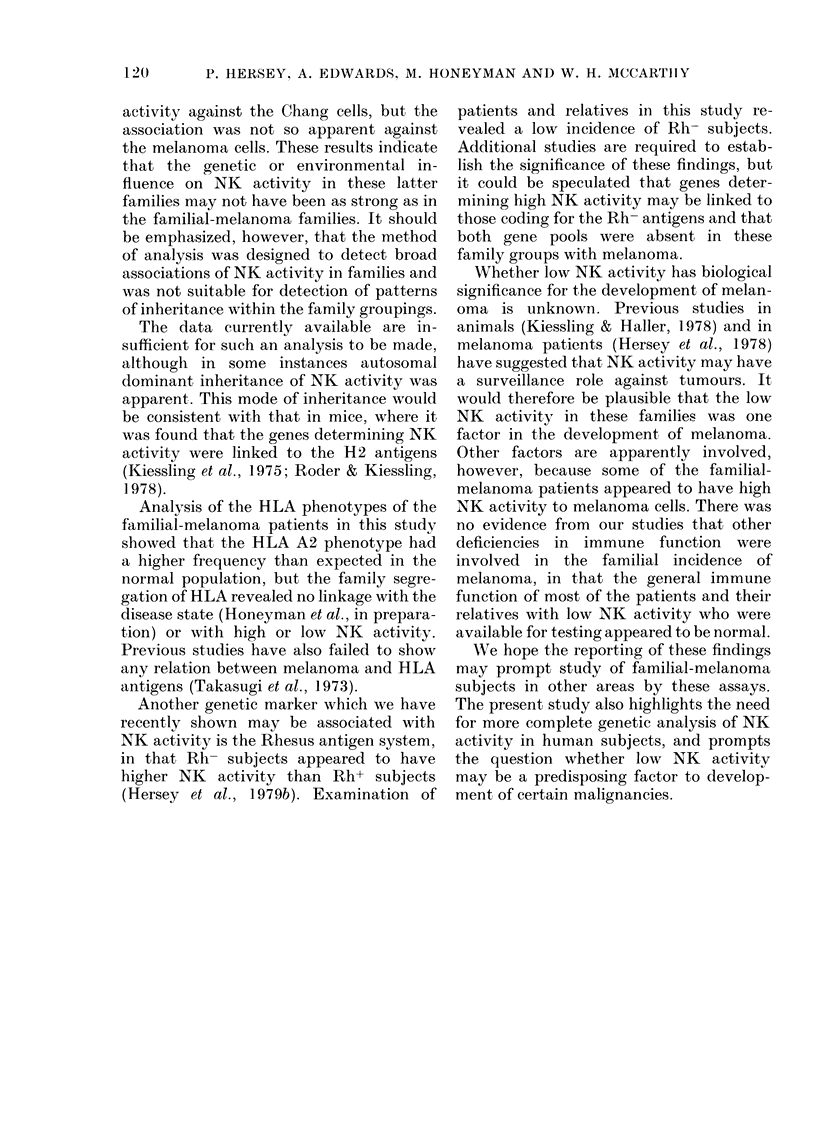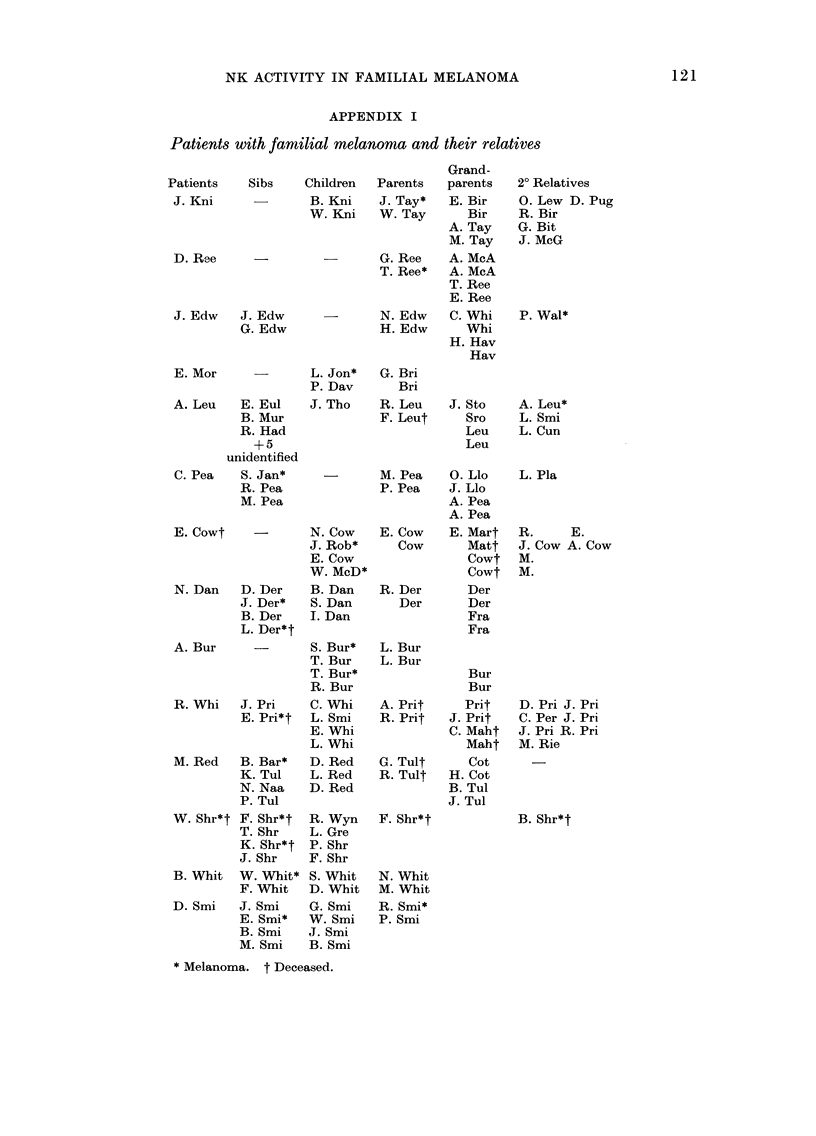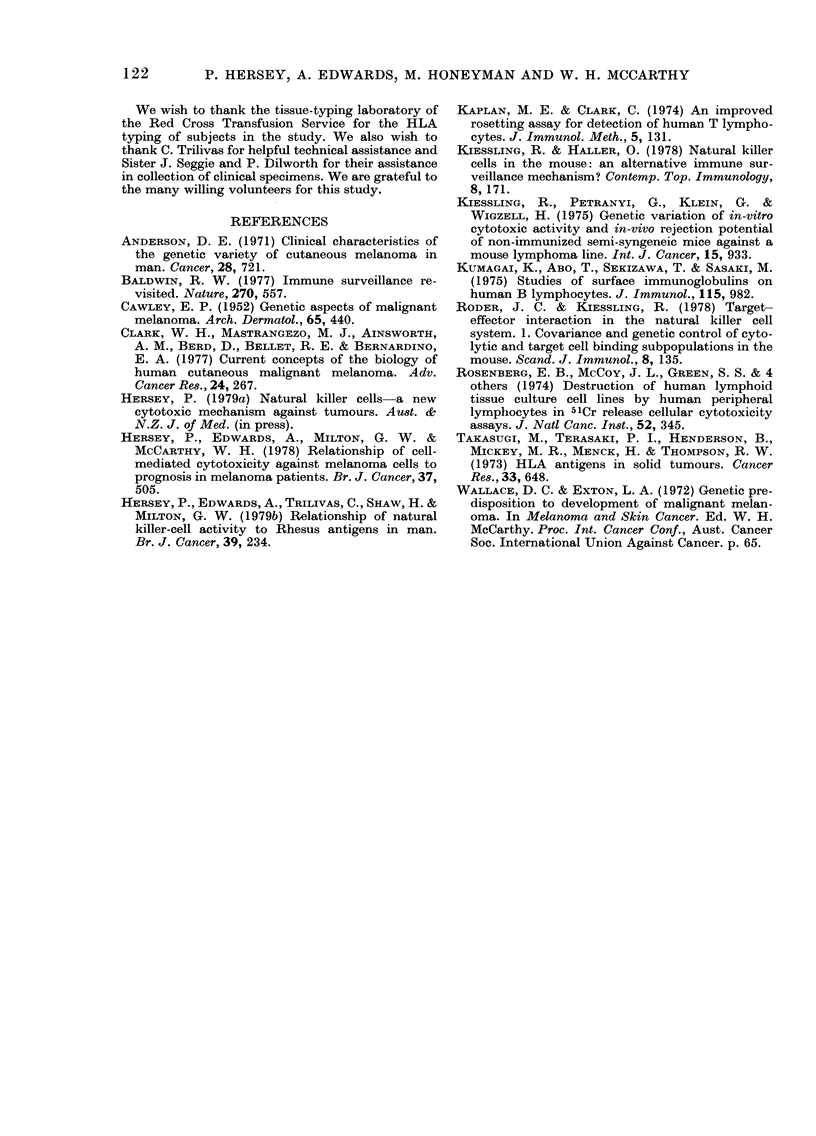# Low natural-killer-cell activity in familial melanoma patients and their relatives.

**DOI:** 10.1038/bjc.1979.147

**Published:** 1979-07

**Authors:** P. Hersey, A. Edwards, M. Honeyman, W. H. McCarthy

## Abstract

Patients with melanoma who had one or more close relatives with melanoma were studied for their natural-killer-cell (NK) activity against cultured melanoma cells and Chang cells. A high proportion of the patients and their relatives were found to have low NK activity against these target cells. In most of the patients this could not be attributed to general depression of their immune function, since B- and T-cell numbers and the mitogenic response to PHA were within normal limits. The levels of NK activity of the patients and their relatives were found to be significantly correlated, suggesting that the NK activity in these families may have been genetically (or environmentally) determined. Several genetic markers were examined in the patients and their relatives for association with the disease state and NK activity. No association with HLA antigens or ABO blood groups was detected, but there was a low incidence of the Rhesus negative phenotype in the patients (the Rh phenotype had previously been associated with high NK activity). The present results indicate that NK activity has a familial association in families with a high incidence of melanoma, and raise the question whether low NK activity may be one of the predisposing factors in the development of familial melanoma.


Br. J. Cancer (1979) 40, 113

### LOW NATURAL-KILLER-CELL ACTIVITY IN FAMILIAL MELANOMA PATIENTS AND THEIR RELATIVES

P. HERSEY, A. EDWARDS, M. HONEYMAN* AND W. H. McCARTHY†

*From the Kanematsu Memorial Institute and Melanoma Unit, †Department of Surgery, University of Sydney at Sydney Hospital, and the *Red Cross Transfusion Service, Sydney, Australia*

Received 3 January 1979 Accepted 12 March 1979

**Summary.—Patients with melanoma who had one or more close relatives with melanoma were studied for their natural-killer-cell (NK) activity against cultured melanoma cells and Chang cells. A high proportion of the patients and their relatives were found to have low NK activity against these target cells. In most of the patients this could not be attributed to general depression of their immune function, since B- and T-cell numbers and the mitogenic response to PHA were within normal limits. The levels of NK activity of the patients and their relatives were found to be significantly correlated, suggesting that the NK activity in these families may have been genetically (or environmentally) determined. Several genetic markers were examined in the patients and their relatives for association with the disease state and NK activity. No association with HLA antigens or ABO blood groups was detected, but there was a low incidence of the Rhesus negative phenotype in the patients (the Rh phenotype had previously been associated with high NK activity).**

**The present results indicate that NK activity has a familial association in families with a high incidence of melanoma, and raise the question whether low NK activity may be one of the predisposing factors in the development of familial melanoma.**

A NUMBER OF REPORTS have confirmed that certain families have a higher incidence of melanoma than that expected in the general population, and that this appears to be attributable to hereditary factors (Cawley, 1952; Anderson, 1971; Wallace & Exton, 1972). Patients in these families tend to develop melanoma at an earlier age, have a higher frequency of multiple melanomas and a greater incidence of other malignancies. The nature of the factors predisposing members in these families to develop melanoma and their inheritance is unknown. In most studies the inheritance appeared to be polygenic, but in some families autosomal dominant inheritance was evident (Anderson, 1971; Wallace & Exton, 1972).

One of the factors considered to be important in control of tumour development is surveillance by the immune system. In particular, recent studies have suggested that cells with natural killer activity against tumour cells may constitute an important surveillance mechanism against tumours (Kiessling & Haller, 1978; Hersey, 1979; Baldwin, 1977). This suggestion was supported by recent findings that melanoma patients with low natural-killer (NK) cell activity may have a shorter period to recurrence of melanoma than those with normal or high NK activity (Hersey *et al.*, 1978).

In the present study the NK activity of patients with familial melanoma and their relatives was examined to determine whether this immune function may be involved in the familial occurrence of melanoma. The results suggest that a high proportion of patients with familial melan-

Correspondence to: Dr P. Hersey, Kanematsu Memorial Institute, Medical Research Department, Sydney Hospital, Macquarie Street, Sydney, N.S.W. 2000, Australia.

oma and their relatives had low NK activity which did not appear to be associated with abnormalities in other aspects of immune function.

### MATERIALS AND METHODS

*Patients with familial melanoma and their relatives.*—Thirteen families in which more than 1 patient with melanoma had been documented by histopathological criteria were included in the study (18 patients and 53 relatives). The known member of each family are listed in Appendix I, but not all were available for study. Patients with a history of melanoma are indicated by an asterisk. All were clinically free of melanoma and, with the exception of Whit, had been so for longer than 1 year. All were untreated. Their ages ranged from 18 to 65 (mean 42·5) years. Most of the patients and their relatives were from country areas and repeat tests on many of these subjects were not possible.

*Patients with non-familial melanoma and their relatives.*—For comparative purposes, patients who had previously had melanoma but who were clinically free of melanoma were taken as index subjects for study of their available relatives. Patients were selected on the basis of previous studies to provide a range of NK activity, so that any genetic influence of NK activity could be detected more readily in their relatives. Studies on family groups were carried out on the same day when possible, to minimize the effects of day-to-day variation in the assays. The ages of the patients ranged from 25 to 72 (mean 45·5) years.

*Normal subjects and their relatives.*—Normal blood donors or hospital personnel with high or low NK values were taken as index subjects. All available relatives and the index subjects were tested if possible on the same day. Ages ranged from 21 to 53 (mean 31·1) years.

*Assays of NK activity.*—The cytotoxic activity of blood mononuclear cells against the target cells was determined by $^{51}$Cr-release assays, as described in previous reports (Hersey *et al.*, 1978). The effector cells were mononuclear cells obtained from defibrinated venous blood by centrifugation on Hypaque–Ficoll mixtures. All assays were carried out on blood samples taken the same day.

Target cells were from the MM200 melanoma cell line established from a primary melanoma in the Queensland Institute for Medical Research, and the Chang cultured human liver cell line (Commonwealth Serum Laboratories, Melbourne). $^{51}$Cr-labelling was carried out by incubation with 100 $\mu$Ci of $Na_2$ $^{51}CrO_4$ for 2 h at 37°C. Target cells ($3\times10^3$ in 0·5 ml) were incubated with effector cells ($3\times10^5$ in 0·5 ml) overnight in duplicate $10\times70$mm round-bottomed tubes. Culture medium was RPMI plus 10% foetal bovine serum FBS (Batch 64, Australian Laboratory Services).

Percent $^{51}$Cr release was calculated as previously described. All results were expressed in terms of percent $^{51}$Cr release above baseline release from the target cells alone. To assess the day-to-day variation in the assays, a standard NK donor was used in each assay from cells stored in liquid $N_2$ from the one donor. All assays were carried out by the one operator (A.E.) which we consider to be an important factor in reduction of day-to-day variability in the assays.

*E rosettes.*—These were carried out by the method of Kaplan & Clark (1974), using aminoethylisothiouronium bromide (AET) treated sheep red blood cells (SRCB). 200 $\mu$l of 1% AET-SRBC and 200 $\mu$l of a suspension of blood mononuclear cells of $2\times10^6$ ml were mixed and incubated at 37°C for 15 min. They were centrifuged at 300 *g* for 5 min and incubated at 4°C for 1 h.

*Mitogenic response to PHA.*—$10^5$ mononuclear cells in 200 $\mu$l of RPMI+10% FBS were cultured for 3 days in 0, 5 or 20 $\mu$g of PHA-P (Difco). Cell division was assessed at this time by the addition of 2 $\mu$Ci of $^{125}$I-iododeoxyuridine ($^{125}$IUDR) for 4 h and then harvested by washing the cells twice in saline and twice in 5% trichloracetic acid.

*B lymphocytes.*—Cells with surface immunoglobulin were detected by use of fluorescein-labelled polyvalent sheep anti-human immunoglobulin (Wellcome reagents). $5\times10^5$ lymphocytes were exposed to acetate buffer at pH 4·5 for 1 min at 4°C to remove non-specifically bound surface immunoglobulins (Kumagai *et al.*, 1975), then washed in phosphate-buffered saline (PBS). The cells in 50 $\mu$l PBS were incubated in 100 $\mu$l of a 1-in-8 dilution of the antiserum for 20 min at room temperature and then washed $\times3$ in PBS. They were then mounted in PBS: glycerol, pH 8·2, and examined by fluorescent microscopy.

*Statistical analysis.*—The relationship between NK values of patients and their first-degree relatives was tested for significance by $t$ test of the correlation coefficient of the values, using the formula $t=r\sqrt{n-2}/1\text{-}r^2$. The significance of the difference in the proportion of familial melanoma patients with low NK activity compared to non-familial melanoma patients was estimated by Chi-square tests and Fisher's exact probability test of the data. The relationship of low NK activity to particular HLA antigens and Rhesus antigens was also estimated by Chi-square test of the data.

### RESULTS

### 1. *Reproducibility of the assays*

The NK activity of the stored mononuclear cells from the control donor was tested on 14 occasions in parallel assays with those carried out on mononuclear cells from subjects belonging to the familial melanoma families. The mean NK activity of the stored cells against the MM200 target cell was 7·8±1·9 (s.d.). Against the Chang cells, equivalent values were 7·8±2·2. To illustrate further the reliability of the assays, the NK values for 2 laboratory donors from repeated tests over a 12-month period corresponding to that of the studies on the familial melanoma patients against the MM200 target cell were recorded. In 10 assays on these 2 normal individuals the means of % $^{51}$Cr release were 13·6±1·5 and 29·9±2·0.

These results indicate that values obtained by single tests on a subject were likely to give a reliable indication of the inherent NK activity of that person, and did not merely reflect chance variation in the assays. When repeat assays were available on the subjects in the study, there was little variation between the tests, as shown in the following tables. Similar degrees of variation in repeated tests on the same individual were reported by Rosenberg *et al.* (1974).

### 2. *Natural cell-mediated cytotoxicity (NK activity) of familial melanoma patients and their relatives*

The NK activity of blood mononuclear cells from patients and available family members against the MM200 and Chang target cells at a ratio of 100:1, effector: target cell, is shown in Table I. The mean NK value of 80 normal subjects was 14·97±5·78 for the MM200 target cell (Hersey *et al.*, 1978) and 14·68±6·2 against the Chang target cell (Hersey *et al.*, 1979*b*). [This latter value was higher than the mean value of 9·6% recorded in Hersey *et al.* (1978). We attribute this to the use of a different line of Chang cells supplied to us from CSL, Mebourne, before the present study).] Based on these data. values less than 10% $^{51}$Cr release (*i.e.* less than 1 s.d. below the mean) were considered as "low" and values greater than 20% (*i.e.* greater than 1 s.d. above the mean) as "high". The results in Table I indicate that 12/18 patients in 9/13 families (Shr family excluded) with familial melanoma had low NK values to the MM200 and/or Chang target cell (proportion of total patients=0·67). Two patients in 2 families (Kni and Smi) had high values, and 4 patients in 4 families (Ree, Red, Bur and Dan) had values within the normal range. No patients were available for study in Family Shr, but the values in the relatives are shown. These were not included in the analyses.

The proportion of non-familial melanoma patients with localized melanoma with less than 10% $^{51}$Cr release against the MM200 target cell, measured after surgical removal of melanoma in a study on 74 patients (Hersey *et al.*, 1978) was 0·39 (29 patients). Comparison of the different proportions of familial and non-familial melanoma patients with values less than 10% gave a value of 5·39 by Chi-squared test ($0{\cdot}025>P>0{\cdot}01$) and $P=0{\cdot}024$ by Fisher's exact test.

The second point to be noted from the table is that the relatives of patients with familial melanoma also tended to have similar NK values against the 2 target cells (*e.g.* the children in Families Kni and Red also had high NK values, and the relatives of Families Mor, Leu, Pea, Cow, Whi had low NK values). The correlation of the NK values of relatives with those

TABLE I.—*Cell-mediated cytotoxicity of patients and relatives of patients with familial melanoma*

| Family | Target cell† | Patient | Siblings | Children | Parents | Second-degree relatives |
|---|---|---|---|---|---|---|
| Kni | M | 34·5±1·5 | — | 23 | | 11, 9 23 |
| | C | 14·5±2·5 | | 16 | | 9, 16, 19 |
| Edw | M | 5 | 10, 14 | — | 20. 30 | — |
| | C | 3 | 10, 13 | | 10. 8 | |
| Mor | M | 3 | — | 10, 5, 7* | | — |
| | C | 6 | — | 12, 4, 6 | | |
| Leu | M | 3 | 7·5±1·4 | | | |
| | | | 4±0, 10±1·6 | 14 | | 12, 29 |
| | C | 9 | 9±0, 9·5±1·4 | — | | 4, 20 |
| | | | 9±0 | | | |
| Pea | M | 3 | 6, 5* | 4 | 6, 6 | 11 |
| | C | 5 | 6, 6 | 4 | 6, 6 | 10 |
| Dan | M | 4 | 13* | 9, 15, 14 | | |
| | C | 1 | 10 | 3, 8, 6 | | |
| Shr | M | | 8·3±1·5, 8±2 | | | |
| | | | 5, 0 | 0 | | |
| | C | | 8, 10 | | | |
| | | | 9·5±1·4, 14·5±2·2 | | | |
| Bur | M | 11 | — | 12*, 15, 10, 3 | 16 | |
| | C | 7 | | 10, 18, 10, — | 7 | |
| Cow | M | 6 | — | 6, 9 | 30 | 26 |
| | C | 6 | | 10. 6 | 20 | 20 |
| Ree | M | 20 | — | 20 | 20 | |
| | C | 13 | | 9, 9±5 | 9 | |
| Whi | M | 9±1·8 | 5±0, 8·5±1·8* | 8±0, 6±0, 8·5±0·5 | | 9, 5, 10, 7 |
| | C | 5±0 | 5±0, 8±2 | 8·5±1·6, 8±1·0 | | 10, 5, 17, 4 |
| Red | M | 18±2 | | 16±1·6, 21·5±2·5 | | |
| | C | 14±0 | | 14±2, 8±0, 12±2 | — | — |
| Whit | M | 5·3±1·4 | | | 4 | |
| | C | 7·3±1·8 | | | 6 | |
| Smi | M | 25 | | | | |
| | C | 10 | | | | |

† M=MM200 and C=Chang target cells.
* Relatives with melanoma. Values given are % $^{51}Cr$ release above baseline $^{51}Cr$ release from target cells alone at a ratio of 100:1 effector:target cells. Spontaneous release for both target cells ranged from 23 to 42%. Standard errors (s.e.) of single tests were <2%. Where 2 or more tests were carried out the s.e. are shown.

of the familial melanoma patients is shown in the Figure. Whit and Smi were not included in this analysis because relatives were not available for study. The correlation coefficient ($r$) examined by $t$ test was highly significant (a) against the MM200 target cell ($r=0{\cdot}48$, $0{\cdot}005>P>0{\cdot}001$) and significant (b) against the Chang target cell ($r=0{\cdot}34$, $0{\cdot}05>P>0{\cdot}025$). The correlation of the NK activity of familial melanoma patients with that of their distant relatives (cousins and uncles) was also examined as shown in Figure (c), but no significant correlation was found ($r=0{\cdot}17$).

### 3. *NK activity of non-familial melanoma patients and their relatives*

The NK activity of 15 melanoma patients and their close relatives studied over the same period is shown in Table II. The purpose of this study was to determine whether the NK activity of their relatives would be similar to that of the patient, and patients were therefore selected to provide a range of high, nor-

FIG.—Correlation of NK activity of familial-melanoma patients with that of their relatives. (a) MM200 target cell; $r=0 \cdot 48$ ($0 \cdot 005>P>0 \cdot 001$) (b) Chang target cell; $r=0 \cdot 34$ ($0 \cdot 05>P>0 \cdot 025$) (c) NK activity against Chang and MM200 target cells of patients compared to that of their distant relatives ($r=0 \cdot 17$, N.S.).

mal and low NK values. There was some evidence that NK values of relatives did correlate with those of the patients. For example, Patients Heb, Ral, Lev and their close relatives had low NK values; Patients Buc, Che and Sla and their relatives tended to have high NK values; and Patients Law, Fen, Pom and their relatives had normal NK values. The one exception was Patient Ben, with high NK values that were not reflected in that of the relatives. The correlation coefficient between patients and their relatives was significant for NK activity against the Chang cell ($r=0 \cdot 29$, $0 \cdot 05>P>0 \cdot 025$) but not against the MM200 target cell ($r=0 \cdot 22$, $0 \cdot 10>P>0 \cdot 05$).

4. *NK activity of normal subjects and their relatives*

Similar studies were carried out on the families of 12 normal subjects to determine whether a familial association of NK activity could be deteted in normal subjects. Index subjects were again selected with high, low and normal NK values. The results in Table III again suggest that NK values of relatives tended to be similar. Correlation coefficient against the MM200 was 0·14 (N.S.) but against the Chang cell was 0·38 ($0 \cdot 02>P>0 \cdot 01$). Unfortunately the data are insufficient to detect whether a particular pattern of inheritance of NK activity in these families (*e.g.* with Family Ayl, if the NK value of the missing parent were high, genes from this parent might have been responsible for the high NK values of the other sib. Similarly, with Family Mur one sib had high and the other low NK values against the target cells. One parent had low values and it would have been helpful to know the NK value of the other parent.

5. *General immune function of patients with familial melanoma and their relatives*

The E rosette (T cell) and surface immunoglobulin (B cell) percentages in peripheral blood, and the mitogenic response to PHA in patients and relatives of 7 of the melanoma families are shown in Table IV. Mean values ±s.d. on 26 normal controls carried out over the same period were 61±11 for E rosettes and 18±7 for surface immunoglobulin-bearing cells. For PHA at concentrations of 0, 5 and 20 $\mu$g these values were 800±600, 8230±4200, and 9100±5300 ct/min. Some members of the Families Pea and Shr had low PHA responses, and one patient Mor

TABLE II.—*Cell-mediated cytotoxicity of non-familial melanoma patients and their relatives (Symbols as in Table I)*

| Family | Target cell | Patient | Siblings | Children | Parents |
|---|---|---|---|---|---|
| Jay | M | 12·6±2·0 | 7·5±1·6 | 7±0·5, 13 | |
| | C | 12±1·7 | 4±2 | 5±0, 25 | |
| Heb | M | 9·9±1·9 | 5·5±0·6 | | 7·5±1·0<br>6±0 |
| | C | 7·7±1·7 | 4±1 | | 5±0<br>6±0 |
| Yat | M | 31·3±2·9 | 12±2 | | 18±3<br>12±2 |
| | C | 7·3±1·5 | 6±1 | | 8·5±2<br>7±1·2 |
| Ben | M | 31±3 | 8, 15, 9±1·4 | 14±1·0 | |
| | C | 27·3±1·8 | 6, 9, 7±1·4 | 13 | |
| Law | M | 15±1·5 | 16 | 15, 6 | |
| | C | 4±1·0 | 9 | 11, 5 | |
| Ral | M | 8±1·7 | 8, 4, 4 | | |
| | C | 13±2 | 15, 9, 10 | | |
| Buc | M | 23±2·5 | 22 | 13 | |
| | C | 8±0·6 | 6 | 6 | |
| Che | M | 22±2 | 7, 22 | | 40, 38 |
| | C | 21±2·6 | 7, 11 | | 20, 11 |
| Lev | M | 9±1·8 | 3 | | 4, 1 |
| | C | 9±1·8 | 16 | | 14, 9 |
| Sla | M | 18±1·8 | 50 | | 15, 30 |
| | C | 18±1·4 | 35 | | 25, 22 |
| Tay | M | 7±1·4 | 10, 14 | — | 16, 36 |
| | C | 11±1·5 | 24, 27 | | 21, 28 |
| Fen | M | 15±2·2 | 20, 10, 11 | — | |
| | C | 11±1·7 | 16, 10, 7 | | |
| San | M | 6±1·2 | | 4·0, 17±1·2 | |
| | C | 4·3±1·2 | | 14, 8±0·6 | |
| Gre | M | 17±0 | | 12 | 2, 2 |
| | C | 8±2 | | 11 | 3, 3 |
| Pom | M | 17 | 20, 33 | 18, 17, 17 | 18, 16 |
| | C | 18 | 20, 20 | 21, 22, 25 | 18, 17 |

See footnotes to Table I. S.e. calculated from at least 4 repeat tests.

had low E-rosette values, but all other patients and relatives appeared to have normal values. Most of the members of these families had low NK activity (see Table I).

### DISCUSSION

The incidence of familial melanoma has been reported to be from 1 to 6% of all melanoma patients (Clark *et al.*, 1977; Anderson, 1971). However, only 18 such patients were available for the present study in this unit, and it is apparent that caution is needed in assessing the significance of studies on such small numbers of patients. Nevertheless on a statistical basis our studies suggested that familial-melanoma patients had a lower NK activity against cultured melanoma and Chang target cells than non-familial-melanoma patients. In addition it was also apparent that high or low NK values in the familial-melanoma patients were reflected in close relatives but not in distant relatives.

These findings were consistent with a genetic or environmental influence on NK activity in these families, which was diluted in the distant relatives. Analysis of the NK activity in normal families and non-familial-melanoma families also revealed a familial association of NK

TABLE III.—*Cell-mediated cytotoxicity of normal family members*

| Family | Target cell | Index subject | Siblings | Children | Parents | 2nd-Degree relatives |
|---|---|---|---|---|---|---|
| Ayl | M | 4 | 28 | 20, 14 | 7 | |
| | C | 6 | 16 | 21, 11 | 7 | |
| Cur | M | 21 | 7, 20 | — | 16, 11 | |
| | C | 19 | 10, 15 | | 30, 23 | |
| Don | M | 6 | 12 | | 13 | |
| | C | 8 | 15 | | 11 | |
| Gill | M | 7 | 5, 15, 19 | 18 | — | |
| | C | 9 | 12, 6, 8 | 11 | | |
| Tho | M | 7 | | 14, 11 | 6, 14 | |
| | C | 11 | | 10, 2 | 3, 12 | |
| Wot | M | 24±0 | — | — | 21, 12 | 8, 16 |
| | C | 18±0·8 | | | 20, 10 | 8, 14 |
| Dil | M | 17 | 9, 8 | | | |
| | C | 8 | 6, 8 | | | |
| Ash | M | 8 | 6 | | 9 | |
| | C | 14 | 7 | | 16 | |
| Gar | M | 10±0·5 | 2, 18 | — | 6, 11 | |
| | C | 9±1·1 | 0, 15 | | 2, 15 | |
| Mur | M | 29·9±2·0 | 5 | 25 | 8 | |
| | C | 6±1·8 | 2 | 18 | 3 | |
| Her | M | 7·7±1·3 | | 5, 2, 2 | | |
| | C | 6·7±1·4 | | 18, 23, 10 | | |
| Kra | M | 9 | 4, 10 | | 3 | |
| | C | 15 | 10, 16 | | 12 | |

See footnotes to Table I.

TABLE IV.—*General immune function of familial-melanoma patients and their relatives*

| Subjects | % E rosettes | % Ig+ cells | PHA response ct/min 0 | 5 μg | 20 μg |
|---|---|---|---|---|---|
| F. Shr (c) | 68 | — | 1670 | 7020 | 7147 |
| P. Shr (c) | 54 | — | 748 | 2072 | 5041 |
| R. Wyn (c) | 72 | — | 443 | 1742 | 3458 |
| L. Gre (c) | 50 | — | 489 | 1967 | 2536 |
| J. Edw (p) | 50 | 28 | 1868 | 6478 | 4514 |
| N. Edw (m) | 58 | 20 | 1350 | 4968 | 4958 |
| J. Edw (b) | 60 | 22 | 629 | 15290 | 13534 |
| G. Edw (b) | 56 | 26 | 635 | 7672 | 7366 |
| E. Mer (p) | 42 | 25 | 855 | 6942 | 8185 |
| N. Dan (p) | 50 | 32 | 1590 | 3880 | 6188 |
| S. Dan (c) | 70 | 26 | 1660 | 6627 | 13940 |
| B. Dan (c) | 52 | 20 | 1330 | 11550 | 15300 |
| I. Dan (c) | 62 | 18 | 1100 | 5807 | 7670 |
| A. Leu (p) | 60 | 20 | 1264 | 2243 | 5308 |
| J. Tho (c) | 72 | 25 | 1472 | 7590 | 10509 |
| E. Enl (s) | 70 | — | 703 | 6102 | 6892 |
| B. Murr (s) | 52 | 25 | 1140 | 10460 | 6070 |
| A. Bur (p) | 56 | 28 | 720 | 11060 | 14500 |
| R. Bur (c) | 60 | 32 | 530 | 10285 | 12893 |
| T. Bur (c) | 68 | 22 | 940 | 7174 | 13330 |
| T. Bur (c) | 60 | 32 | 1644 | 12520 | 15500 |
| L. Bre (m) | 58 | 36 | 1170 | 14803 | 15040 |
| C. Pea (p) | 60 | 18 | 396 | 2130 | 3620 |
| S. Jan (p) | 66 | 26 | 505 | 2300 | 3804 |
| P. Pea (m) | 68 | — | 550 | 2275 | 3260 |

(p), (m), (c), (s) and (b) = patient, mother, child, sister and brother respectively.

activity against the Chang cells, but the association was not so apparent against the melanoma cells. These results indicate that the genetic or environmental influence on NK activity in these latter families may not have been as strong as in the familial-melanoma families. It should be emphasized, however, that the method of analysis was designed to detect broad associations of NK activity in families and was not suitable for detection of patterns of inheritance within the family groupings.

The data currently available are insufficient for such an analysis to be made, although in some instances autosomal dominant inheritance of NK activity was apparent. This mode of inheritance would be consistent with that in mice, where it was found that the genes determining NK activity were linked to the H2 antigens (Kiessling *et al.*, 1975; Roder & Kiessling, 1978).

Analysis of the HLA phenotypes of the familial-melanoma patients in this study showed that the HLA A2 phenotype had a higher frequency than expected in the normal population, but the family segregation of HLA revealed no linkage with the disease state (Honeyman *et al.*, in preparation) or with high or low NK activity. Previous studies have also failed to show any relation between melanoma and HLA antigens (Takasugi *et al.*, 1973).

Another genetic marker which we have recently shown may be associated with NK activity is the Rhesus antigen system, in that $Rh^-$ subjects appeared to have higher NK activity than $Rh^+$ subjects (Hersey *et al.*, 1979*b*). Examination of patients and relatives in this study revealed a low incidence of $Rh^-$ subjects. Additional studies are required to establish the significance of these findings, but it could be speculated that genes determining high NK activity may be linked to those coding for the $Rh^-$ antigens and that both gene pools were absent in these family groups with melanoma.

Whether low NK activity has biological significance for the development of melanoma is unknown. Previous studies in animals (Kiessling & Haller, 1978) and in melanoma patients (Hersey *et al.*, 1978) have suggested that NK activity may have a surveillance role against tumours. It would therefore be plausible that the low NK activity in these families was one factor in the development of melanoma. Other factors are apparently involved, however, because some of the familial-melanoma patients appeared to have high NK activity to melanoma cells. There was no evidence from our studies that other deficiencies in immune function were involved in the familial incidence of melanoma, in that the general immune function of most of the patients and their relatives with low NK activity who were available for testing appeared to be normal.

We hope the reporting of these findings may prompt study of familial-melanoma subjects in other areas by these assays. The present study also highlights the need for more complete genetic analysis of NK activity in human subjects, and prompts the question whether low NK activity may be a predisposing factor to development of certain malignancies.

APPENDIX I

*Patients with familial melanoma and their relatives*

| Patients | Sibs | Children | Parents | Grand-parents | 2° Relatives |
|---|---|---|---|---|---|
| J. Kni | — | B. Kni<br>W. Kni | J. Tay*<br>W. Tay | E. Bir<br>Bir<br>A. Tay<br>M. Tay | O. Lew D. Pug<br>R. Bir<br>G. Bit<br>J. McG |
| D. Ree | — | — | G. Ree<br>T. Ree* | A. McA<br>A. McA<br>T. Ree<br>E. Ree | |
| J. Edw | J. Edw<br>G. Edw | — | N. Edw<br>H. Edw | C. Whi<br>Whi<br>H. Hav<br>Hav | P. Wal* |
| E. Mor | — | L. Jon*<br>P. Dav | G. Bri<br>Bri | | |
| A. Leu | E. Eul<br>B. Mur<br>R. Had<br>+5<br>unidentified | J. Tho | R. Leu<br>F. Leu† | J. Sto<br>Sro<br>Leu<br>Leu | A. Leu*<br>L. Smi<br>L. Cun |
| C. Pea | S. Jan*<br>R. Pea<br>M. Pea | — | M. Pea<br>P. Pea | O. Llo<br>J. Llo<br>A. Pea<br>A. Pea | L. Pla |
| E. Cow† | — | N. Cow<br>J. Rob*<br>E. Cow<br>W. McD* | E. Cow<br>Cow | E. Mar†<br>Mat†<br>Cow†<br>Cow† | R. E.<br>J. Cow A. Cow<br>M.<br>M. |
| N. Dan | D. Der<br>J. Der*<br>B. Der<br>L. Der*† | B. Dan<br>S. Dan<br>I. Dan | R. Der<br>Der | Der<br>Der<br>Fra<br>Fra | |
| A. Bur | — | S. Bur*<br>T. Bur<br>T. Bur*<br>R. Bur | L. Bur<br>L. Bur | Bur<br>Bur | |
| R. Whi | J. Pri<br>E. Pri*† | C. Whi<br>L. Smi<br>E. Whi<br>L. Whi | A. Pri†<br>R. Pri† | Pri†<br>J. Pri†<br>C. Mah†<br>Mah† | D. Pri J. Pri<br>C. Per J. Pri<br>J. Pri R. Pri<br>M. Rie |
| M. Red | B. Bar*<br>K. Tul<br>N. Naa<br>P. Tul | D. Red<br>L. Red<br>D. Red | G. Tul†<br>R. Tul† | Cot<br>H. Cot<br>B. Tul<br>J. Tul | — |
| W. Shr*† | F. Shr*†<br>T. Shr<br>K. Shr*†<br>J. Shr | R. Wyn<br>L. Gre<br>P. Shr<br>F. Shr | F. Shr*† | | B. Shr*† |
| B. Whit | W. Whit*<br>F. Whit | S. Whit<br>D. Whit | N. Whit<br>M. Whit | | |
| D. Smi | J. Smi<br>E. Smi*<br>B. Smi<br>M. Smi | G. Smi<br>W. Smi<br>J. Smi<br>B. Smi | R. Smi*<br>P. Smi | | |

* Melanoma. † Deceased.

We wish to thank the tissue-typing laboratory of the Red Cross Transfusion Service for the HLA typing of subjects in the study. We also wish to thank C. Trilivas for helpful technical assistance and Sister J. Seggie and P. Dilworth for their assistance in collection of clinical specimens. We are grateful to the many willing volunteers for this study.

### REFERENCES

ANDERSON, D. E. (1971) Clinical characteristics of the genetic variety of cutaneous melanoma in man. *Cancer*, **28**, 721.

BALDWIN, R. W. (1977) Immune surveillance revisited. *Nature*, **270**, 557.

CAWLEY, E. P. (1952) Genetic aspects of malignant melanoma. *Arch. Dermatol.*, **65**, 440.

CLARK, W. H., MASTRANGEZO, M. J., AINSWORTH, A. M., BERD, D., BELLET, R. E. & BERNARDINO, E. A. (1977) Current concepts of the biology of human cutaneous malignant melanoma. *Adv. Cancer Res.*, **24**, 267.

HERSEY, P. (1979*a*) Natural killer cells—a new cytotoxic mechanism against tumours. *Aust. & N.Z. J. of Med.* (in press).

HERSEY, P., EDWARDS, A., MILTON, G. W. & MCCARTHY, W. H. (1978) Relationship of cell-mediated cytotoxicity against melanoma cells to prognosis in melanoma patients. *Br. J. Cancer*, **37**, 505.

HERSEY, P., EDWARDS, A., TRILIVAS, C., SHAW, H. & MILTON, G. W. (1979*b*) Relationship of natural killer-cell activity to Rhesus antigens in man. *Br. J. Cancer*, **39**, 234.

KAPLAN, M. E. & CLARK, C. (1974) An improved rosetting assay for detection of human T lymphocytes. *J. Immunol. Meth.*, **5**, 131.

KIESSLING, R. & HALLER, O. (1978) Natural killer cells in the mouse: an alternative immune surveillance mechanism? *Contemp. Top. Immunology*, **8**, 171.

KIESSLING, R., PETRANYI, G., KLEIN, G. & WIGZELL, H. (1975) Genetic variation of *in-vitro* cytotoxic activity and *in-vivo* rejection potential of non-immunized semi-syngeneic mice against a mouse lymphoma line. *Int. J. Cancer*, **15**, 933.

KUMAGAI, K., ABO, T., SEKIZAWA, T. & SASAKI, M. (1975) Studies of surface immunoglobulins on human B lymphocytes. *J. Immunol.*, **115**, 982.

RODER, J. C. & KIESSLING, R. (1978) Target–effector interaction in the natural killer cell system. 1. Covariance and genetic control of cytolytic and target cell binding subpopulations in the mouse. *Scand. J. Immunol.*, **8**, 135.

ROSENBERG, E. B., MCCOY, J. L., GREEN, S. S. & 4 others (1974) Destruction of human lymphoid tissue culture cell lines by human peripheral lymphocytes in $^{51}Cr$ release cellular cytotoxicity assays. *J. Natl Canc. Inst.*, **52**, 345.

TAKASUGI, M., TERASAKI, P. I., HENDERSON, B., MICKEY, M. R., MENCK, H. & THOMPSON, R. W. (1973) HLA antigens in solid tumours. *Cancer Res.*, **33**, 648.

WALLACE, D. C. & EXTON, L. A. (1972) Genetic predisposition to development of malignant melanoma. In *Melanoma and Skin Cancer*. Ed. W. H. McCarthy. *Proc. Int. Cancer Conf.*, Aust. Cancer Soc. International Union Against Cancer. p. 65.